# Supplementary material for: Human brain responses are modulated when exposed to optimized natural images or synthetically generated images
Source: Commun Biol. 2023 Oct 23;6:1076. doi: 10.1038/s42003-023-05440-7 (PMC10593916; doi:10.1038/s42003-023-05440-7)
Supplement: Supplementary file 2 — Supplementary Information [file 42003_2023_5440_MOESM2_ESM.pdf]

Supplementary Information for manuscript: *Human brain responses are modulated when exposed to optimized natural images or synthetically generated images*

Zijin Gu,[1] Keith W. Jamison,[2] Mert R. Sabuncu,[1] Amy Kuceyeski [2]*

[1]School of Electrical and Computer Engineering, Cornell University, Ithaca, New York, USA
[2]Department of Radiology, Weill Cornell Medicine, New York, New York, USA
*To whom correspondence should be addressed; E-mail: amk2012@med.cornell.edu.

Supplementary Figures

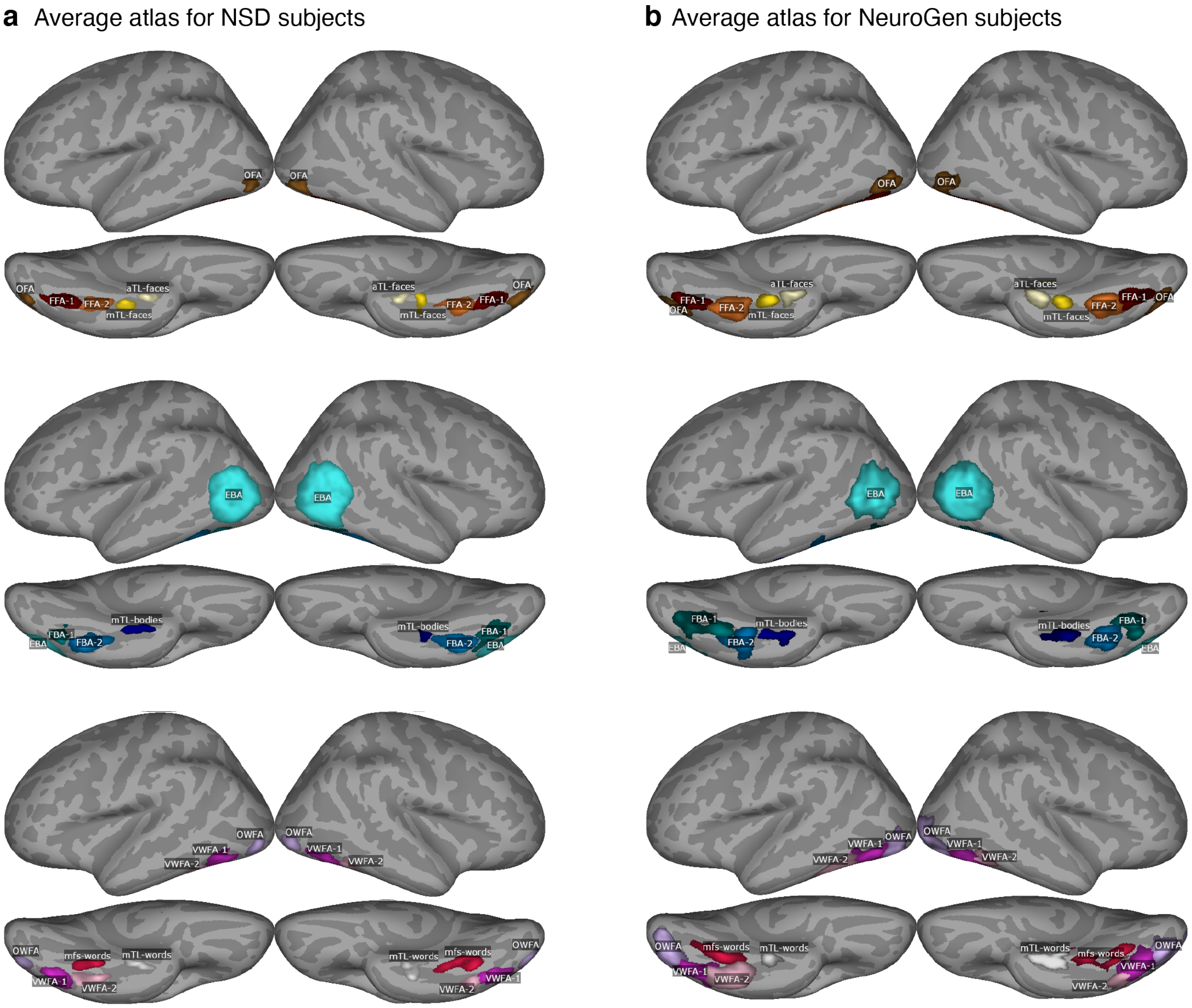

Supplementary Figure 1: Atlas of face, body and word regions. **a** NSD subjects averaged atlas. **b** NeuroGen subjects averaged atlas.

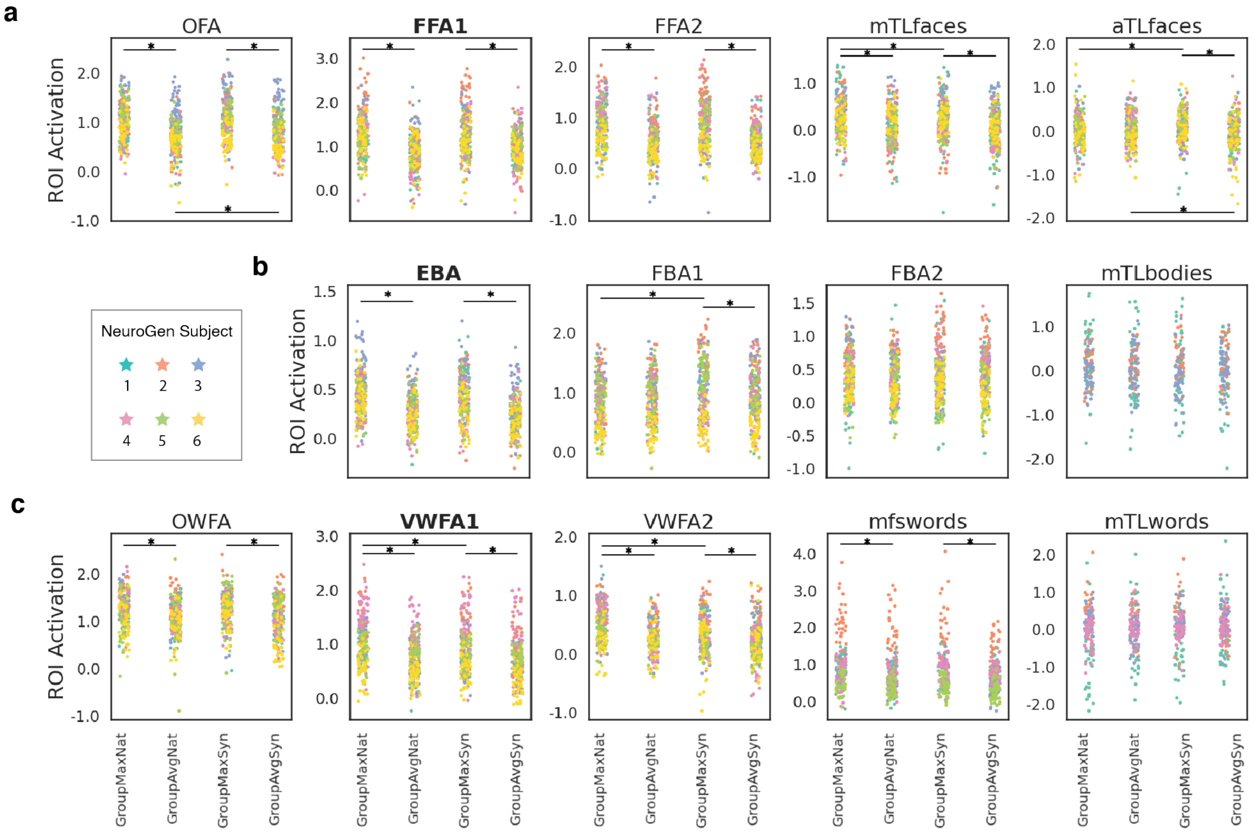

Supplementary Figure 2: Brain activations in response to the "Max" condition images are significantly higher than the activations in response to the "Avg" condition images for their targeted region (and for almost all regions in the same response category), for both natural ("Nat") and synthetic ("Syn") image sets. Scatterplots show the individual observations of the raw fMRI activity in response to the different image conditions in **a** face regions, **b** body regions and **c** word regions. Different subjects are represented by different color points. The following group comparisons were performed via fitting linear mixed effects (LME) models: "GroupMaxNat" vs "GroupAvgNat", "GroupMaxSyn" vs "GroupAvgSyn", "GroupMaxSyn" vs "GroupMaxNat" and "GroupAvgSyn" vs "GroupAvgNat". Significant differences based on permutation testing (FDR corrected $p < 0.05$) are marked with a starred horizontal line.

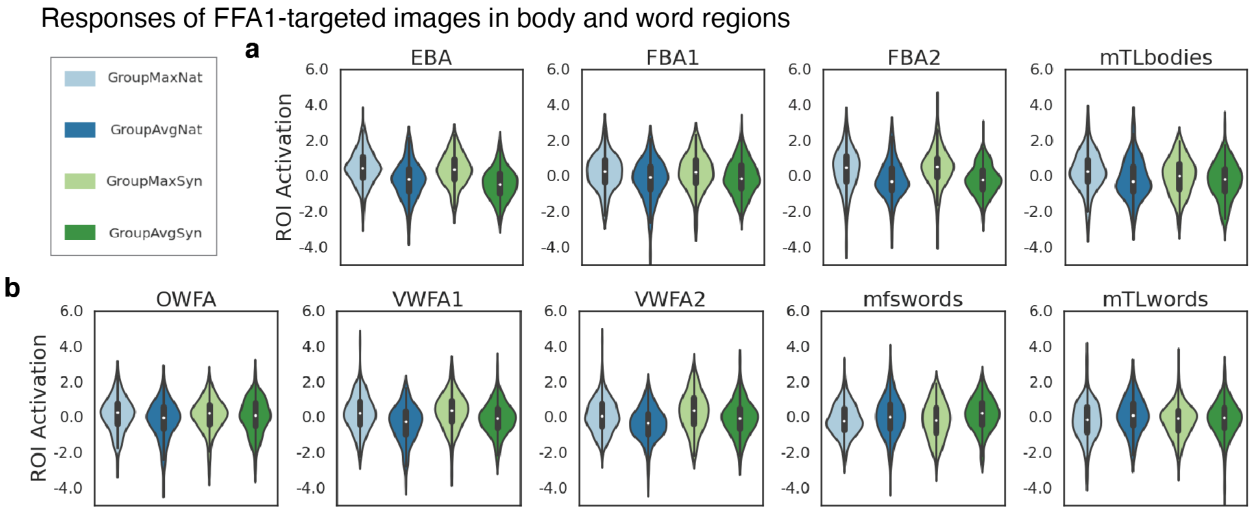

Supplementary Figure 3: Responses of FFA1-targeted images in body and word regions of Session 1. Inside each violin, median is shown by the small white dot in the middle of the boxplot, first quartile and third quartile are indicated by the upper and lower boundary of the black box, and the vertical line shows minimum and maximum. **a** Responses in body regions. **b** Responses in word regions.

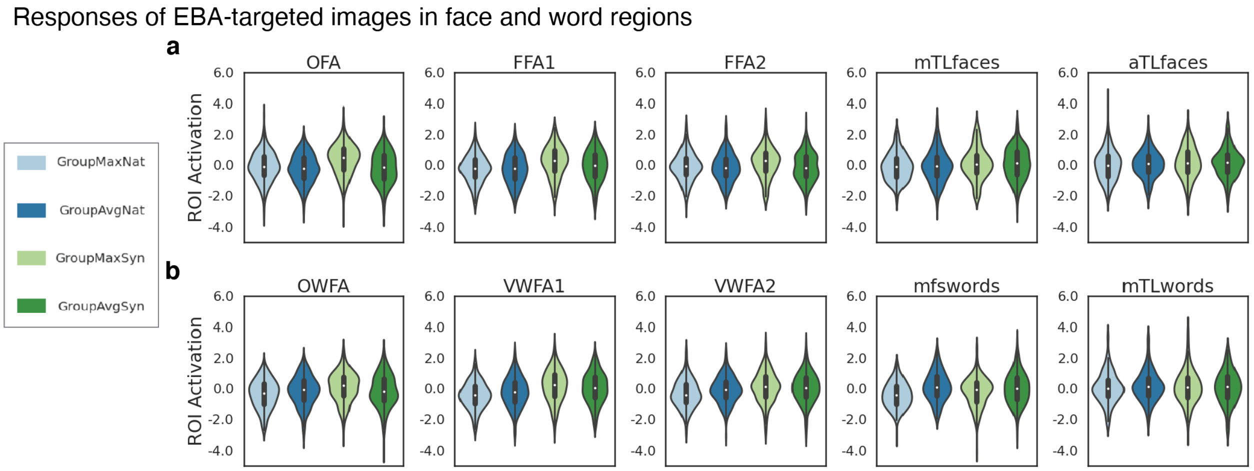

Supplementary Figure 4: Responses of EBA-targeted images in face and word regions of Session 1. Inside each violin, median is shown by the small white dot in the middle of the boxplot, first quartile and third quartile are indicated by the upper and lower boundary of the black box, and the vertical line shows minimum and maximum. **a** Responses in face regions. **b** Responses in word regions.

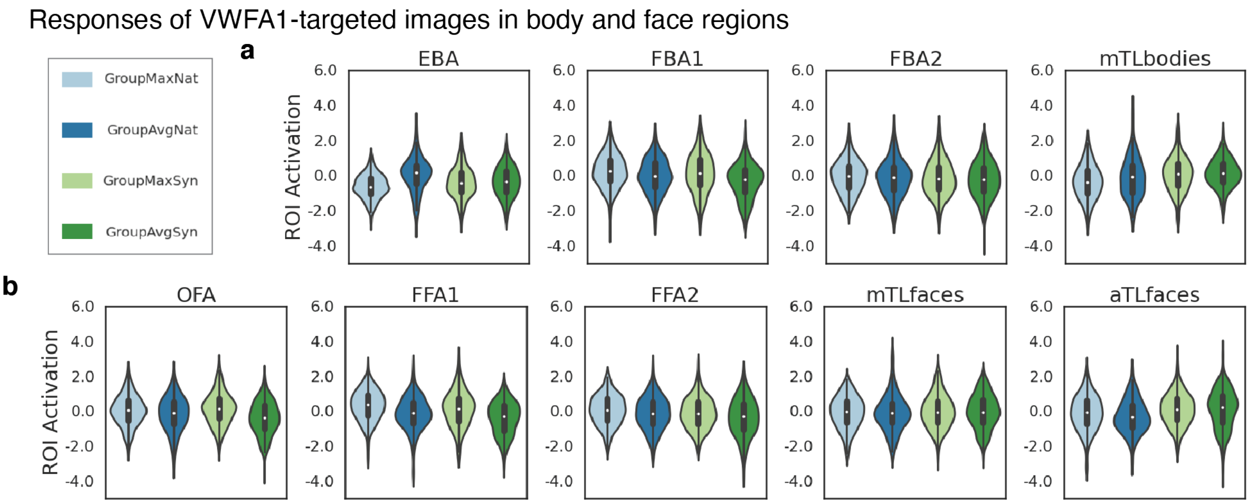

Supplementary Figure 5: Responses of VWFA1-targeted images in body and face regions of Session 1. Inside each violin, median is shown by the small white dot in the middle of the boxplot, first quartile and third quartile are indicated by the upper and lower boundary of the black box, and the vertical line shows minimum and maximum. **a** Responses in body regions. **b** Responses in face regions.

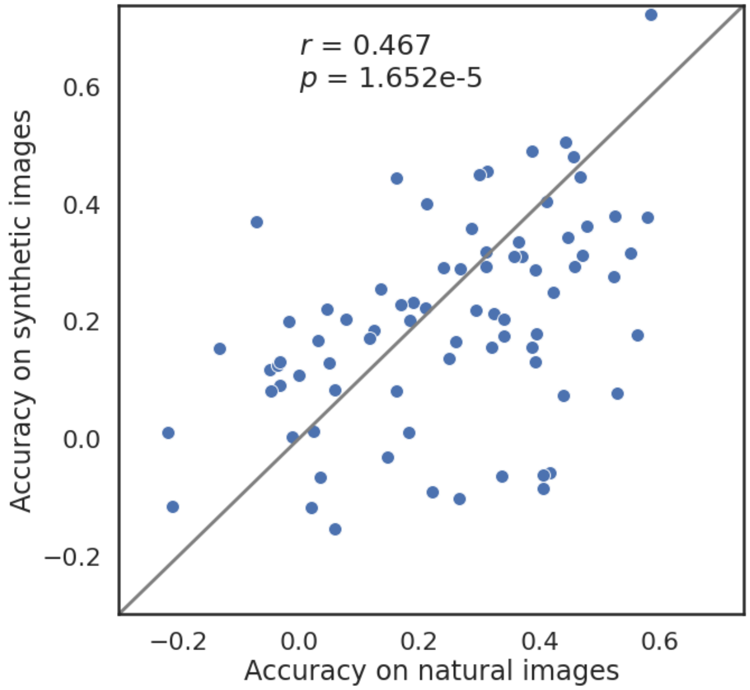

Supplementary Figure 6: Encoding models performed similarly on natural and synthetic images. Accuracy of the NSD-trained "Group" encoding model in predicting the responses of the 6 prospective individuals' brain activities, separately for natural (x-axis) and synthetic (y-axis) images. Note that while it does appear the accuracy may be slightly higher for natural images, generally the accuracies are similar.

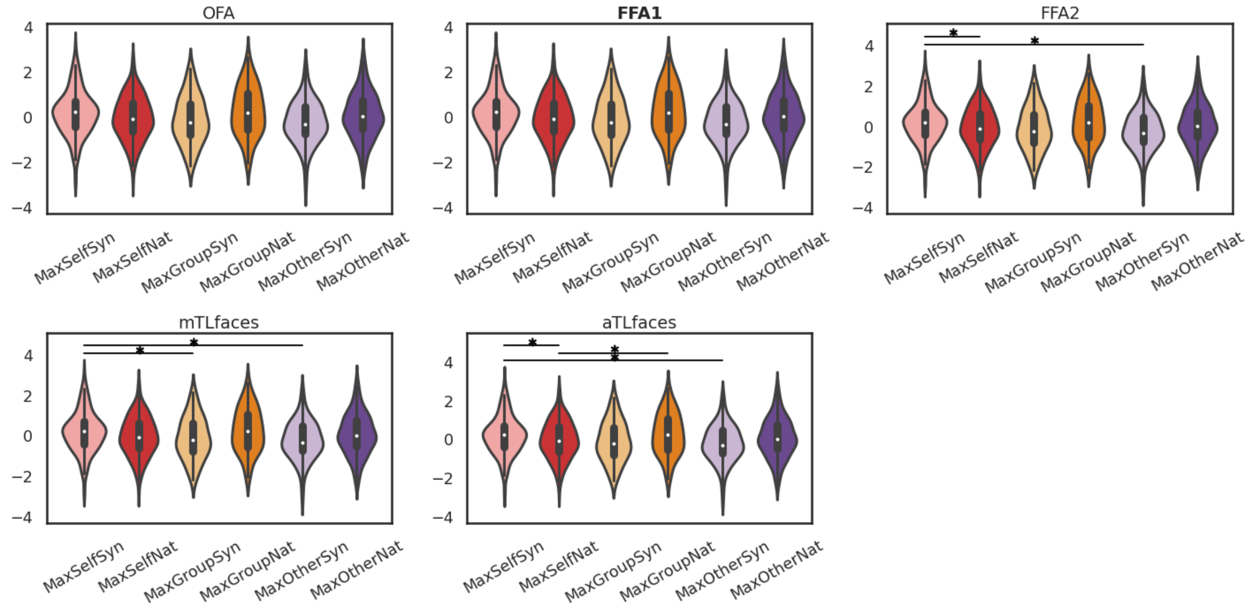

Supplementary Figure 7: Responses of FFA1-targeted images in different face regions of Session 2. Different vilion colors represent different image conditions. Inside each violin, median is shown by the small white dot in the middle of the boxplot, first quartile and third quartile are indicated by the upper and lower boundary of the black box, and the vertical line shows minimum and maximum. The following comparisons were performed via fitting linear mixed effects (LME) models: "MaxGroupSyn" vs "MaxSelfSyn", "MaxOtherSyn" vs "MaxSelfSyn", "MaxGroupNat" vs "MaxSelfNat", "MaxOtherNat" vs "MaxSelfNat", and "MaxSelfNat" vs "MaxSelfSyn". Significant differences based on permutation testing (FDR corrected $p < 0.05$) are marked with a starred horizontal line.

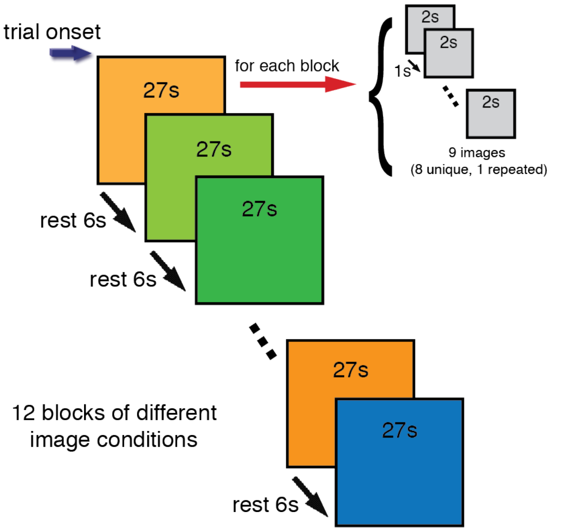

Session 1
12 blocks order example

1	EBA-GroupMaxNat
2	FFA1-GroupMaxNat
3	FFA1-GroupMaxSyn
4	EBA-GroupAvgNat
5	VWFA1-GroupAvgNat
6	FFA1-GroupAvgNat
7	EBA-GroupAvgSyn
8	VWFA1-GroupAvgSyn
9	VWFA1-GroupMaxNat
10	FFA1-GroupAvgSyn
11	EBA-GroupMaxSyn
12	VWFA1-GroupMaxSyn

Session 2
12 blocks order example

1	EBA-OtherMaxSyn
2	VWFA1-SelfMaxNat
3	EBA-SelfMaxNat
4	VWFA1-SelfMaxSyn
5	FFA1-OtherMaxNat
6	FFA1-OtherMaxSyn
7	FFA1-SelfMaxNat
8	EBA-OtherMaxNat
9	FFA1-SelfMaxSyn
10	VWFA1-OtherMaxSyn
11	EBA-SelfMaxSyn
12	VWFA1-OtherMaxNat

Note: actual block orders were randomized each time

Supplementary Figure 8: Experimental design of the fMRI study. During each scan, stimuli were presented for 2s on and 1s off, and were organized into blocks for each condition. 8 unique stimuli were presented per block, with one image repeated in each block for use as a one-back behavioral task. A single 350-second scan consisted of 10 27-second stimulus blocks with 6 seconds of rest between blocks. Each session consisted of 7-10 task scans.

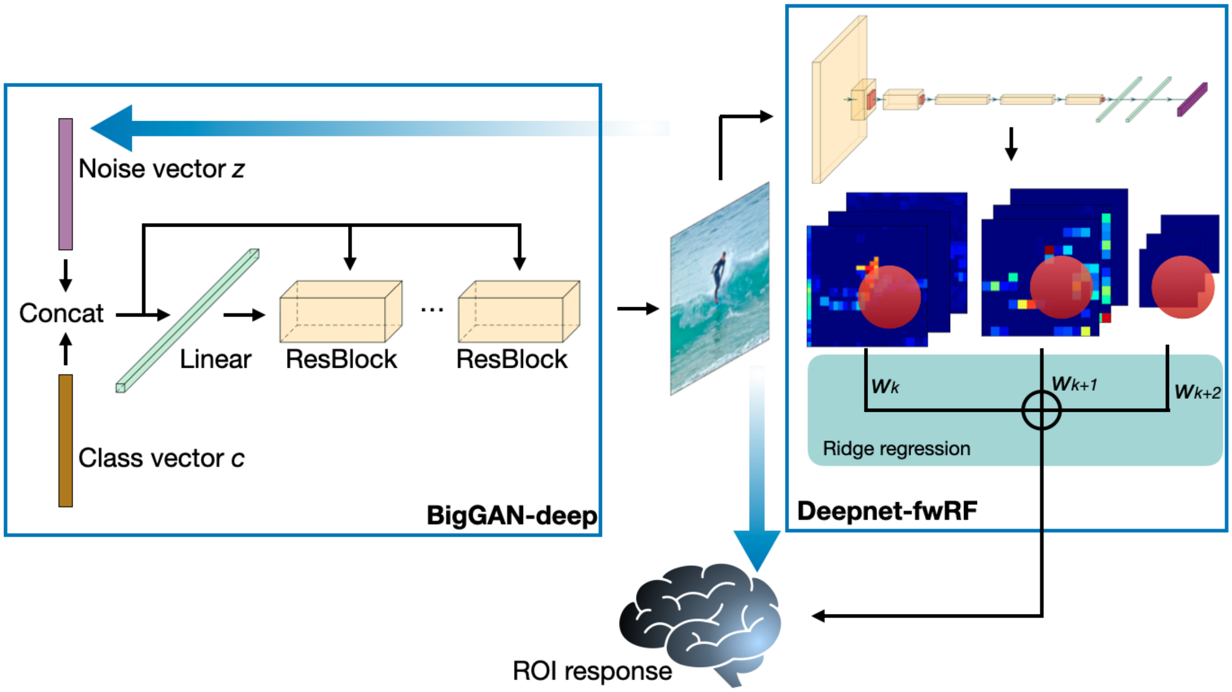

Supplementary Figure 9: The NeuroGen workflow. BigGAN-deep is used as the image generator which takes in a class vector c and a noise vector z and outputs an image. The output image is then fed into the deepnet-fwRF encoding model to get the predicted activation of a region for that image. The class vector is one-hot encoded and kept fixed during the optimization while the noise vector gets updated by minimizing a self-defined loss function, e.g. when maximizing the activation the loss function can be the negative predicted activation. The truncation parameter that control the initialization of the noise vector was set to 0.4 and we run the optimization for 1000 iterations.
