# Supplementary material for: Human brain responses are modulated when exposed to optimized natural images or synthetically generated images
Source: Commun Biol. 2023 Oct 23;6:1076. doi: 10.1038/s42003-023-05440-7 (PMC10593916; doi:10.1038/s42003-023-05440-7)
Supplement: Supplementary file 3 — Description of Additional Supplementary Files [file 42003_2023_5440_MOESM3_ESM.pdf]

Description of Additional Supplementary Files

File name: Supplementary Data 1
Description: Source data behind Figure 2.

File name: Supplementary Data 2
Description: Source data behind Figure 3.
