# Supplementary material for: Human brain responses are modulated when exposed to optimized natural images or synthetically generated images
Source: Commun Biol. 2023 Oct 23;6:1076. doi: 10.1038/s42003-023-05440-7 (PMC10593916; doi:10.1038/s42003-023-05440-7)
Supplement: Supplementary file 6 — Reporting Summary [file 42003_2023_5440_MOESM6_ESM.pdf]

nature portfolio

Corresponding author(s): Amy Kuceyeski

Last updated by author(s): Sep 23, 2023

Reporting Summary

Nature Portfolio wishes to improve the reproducibility of the work that we publish. This form provides structure for consistency and transparency in reporting. For further information on Nature Portfolio policies, see our Editorial Policies and the Editorial Policy Checklist.

Statistics

For all statistical analyses, confirm that the following items are present in the figure legend, table legend, main text, or Methods section.

n/a	Confirmed	
☐	☒	The exact sample size (*n*) for each experimental group/condition, given as a discrete number and unit of measurement
☐	☒	A statement on whether measurements were taken from distinct samples or whether the same sample was measured repeatedly
☐	☒	The statistical test(s) used AND whether they are one- or two-sided<br>*Only common tests should be described solely by name; describe more complex techniques in the Methods section.*
☒	☐	A description of all covariates tested
☐	☒	A description of any assumptions or corrections, such as tests of normality and adjustment for multiple comparisons
☐	☒	A full description of the statistical parameters including central tendency (e.g. means) or other basic estimates (e.g. regression coefficient) AND variation (e.g. standard deviation) or associated estimates of uncertainty (e.g. confidence intervals)
☐	☒	For null hypothesis testing, the test statistic (e.g. F, t, r) with confidence intervals, effect sizes, degrees of freedom and P value noted<br>*Give P values as exact values whenever suitable.*
☒	☐	For Bayesian analysis, information on the choice of priors and Markov chain Monte Carlo settings
☒	☐	For hierarchical and complex designs, identification of the appropriate level for tests and full reporting of outcomes
☐	☒	Estimates of effect sizes (e.g. Cohen's *d*, Pearson's *r*), indicating how they were calculated

Our web collection on statistics for biologists contains articles on many of the points above.

Software and code

Policy information about availability of computer code

Data collection	No software was used for data collection.
Data analysis	Code is available at https://github.com/zijin-gu/neural-modulation.

For manuscripts utilizing custom algorithms or software that are central to the research but not yet described in published literature, software must be made available to editors and reviewers. We strongly encourage code deposition in a community repository (e.g. GitHub). See the Nature Portfolio guidelines for submitting code & software for further information.

Data

Policy information about availability of data

All manuscripts must include a data availability statement. This statement should provide the following information, where applicable:

- Accession codes, unique identifiers, or web links for publicly available datasets
- A description of any restrictions on data availability
- For clinical datasets or third party data, please ensure that the statement adheres to our policy

The Natural Scene Dataset is publicly available at http://naturalscenesdataset.org. The NeuroGen Dataset will is publicly available at https://figshare.com/articles/dataset/NeuroGen_Dataset/23582403.

Research involving human participants, their data, or biological material

Policy information about studies with human participants or human data. See also policy information about sex, gender (identity/presentation), and sexual orientation and race, ethnicity and racism.

Reporting on sex and gender	Natural Scenes Dataset: 6 females, 2 males,<br>NeuroGen Dataset: 5 females, 1 male<br>Sex or gender is not relevant to this study so not considered in the study design and are determined based on self-reporting.
Reporting on race, ethnicity, or other socially relevant groupings	Natural Scenes Dataset: 5 White, 3 Asian, Not Hispanic<br>NeuroGen Dataset: we didn't collect such information as it is not relevant to our study
Population characteristics	Natural Scene Dataset: age 19-32 years<br>NeuroGen Dataset: age 19-25 years<br>All participants are young healthy adults.
Recruitment	Participants were recruited by sending out flyers around the campus and should not contain bias.
Ethics oversight	Institutional Review Board for Human Participant Research

Note that full information on the approval of the study protocol must also be provided in the manuscript.

Field-specific reporting

Please select the one below that is the best fit for your research. If you are not sure, read the appropriate sections before making your selection.

☒ Life sciences ☐ Behavioural & social sciences ☐ Ecological, evolutionary & environmental sciences

For a reference copy of the document with all sections, see nature.com/documents/nr-reporting-summary-flat.pdf

Life sciences study design

All studies must disclose on these points even when the disclosure is negative.

Sample size	The sample size (number of subject) for Natural Scene Dataset is 8 and for NeuroGen is 6.
Data exclusions	No data were excluded from the analysis.
Replication	Replications across subjects and datasets were successful.
Randomization	Randomization is not relevant to this study as samples allocation was not needed.
Blinding	Blinding is not relevant to this study as group allocation was not needed.

Reporting for specific materials, systems and methods

We require information from authors about some types of materials, experimental systems and methods used in many studies. Here, indicate whether each material, system or method listed is relevant to your study. If you are not sure if a list item applies to your research, read the appropriate section before selecting a response.

Materials & experimental systems

n/a	Involved in the study	
☒	☐	Antibodies
☒	☐	Eukaryotic cell lines
☒	☐	Palaeontology and archaeology
☒	☐	Animals and other organisms
☒	☐	Clinical data
☒	☐	Dual use research of concern
☒	☐	Plants

Methods

n/a	Involved in the study	
☒	☐	ChIP-seq
☒	☐	Flow cytometry
☐	☒	MRI-based neuroimaging

Magnetic resonance imaging

Experimental design

Design type	Task functional MRI
Design specifications	The NeuroGen dataset (the novel dataset in this paper, the Natural Scenes Dataset is described elsewhere in full detail) contains MRI data from 6 individuals that consists of two scans about 4 months apart. The task functional MRI collected during both sessions consisted of viewing a series of images that were square cropped and resized to 8.4° x 8.4°. All sessions had the following organization: 3 second inter-stimulus interval, with 2 seconds on, 1 second off. Stimuli were organized into blocks with 8 unique images per block and 1 one-back repeat per block, so 9 stimuli per block = 27 seconds per block. There was a 6 second rest between blocks. Session 1 had 10 runs with 12 blocks each while session 2 had 7 runs with 12 blocks each. A custom PsychoPy script was used to present the stimuli.
Behavioral performance measures	Participants were asked to perform an image recognition task (1-back) to encourage maintenance of attention. No statistics were used to quantify whether the task was performed as expected.

Acquisition

Imaging type(s)	functional MRI, anatomical MRI
Field strength	3T
Sequence & imaging parameters	gradient-echo EPI, 2.25x2.25x3.00mm, 27 interleaved slices, TR=1.45s, TE=32ms, session-encoding in the A»P direction
Area of acquisition	fMRI scans had posterior oblique-axial slices oriented to capture early visual areas and the ventral visual stream

Diffusion MRI ☐ Used ☒ Not used

Preprocessing

Preprocessing software	Preprocessing was done using custom bash and python scripts using FSL tools for motion correction and coregistration, and custom python scripts for slice time correction and temporal upsampling
Normalization	Data were not normalized as we were interested in the individuals' brain responses at a regional level and not group level analysis of voxel-wise data
Normalization template	Data were not normalized
Noise and artifact removal	EPI susceptibility distortion was estimated using pairs of spin-echo scans with reversed session-encoding directions. Preprocessing included slice-timing correction with upsampling to 1 second TR, followed by a single-step spatial interpolation combining motion, distortion, and resampling to 2mm isotropic voxels.
Volume censoring	There was no explicit volume censoring. The single-trial responses were estimated using GLMsingle (https://www.biorxiv.org/content/10.1101/2022.01.31.478431v1, https://github.com/cvnlab/GLMsingle), which constructs data-driven nuisance regressors along with motion time courses to denoise and fit the data

Statistical modeling & inference

Model type and settings	A Generalized Linear Model (GLM) was used to quantify brain activity in response to image presentation. Then the single-trial beta weights representing the voxel-wise response to the image presented was estimated using a GLM. There are three steps for the GLM: the first is to estimate the voxel-specific hemodynamic response functions (HRFs); the second is to apply the GLMdenoise technique to the single-trial GLM framework; and the third is to use an efficient ridge regression to regularize and improve the accuracy of the beta weights, which represent activation in response to the image. FreeSurfer was used to reconstruct the cortical surface, and both volume- and surface-based versions of the voxel-wise response maps were created.
Effect(s) tested	The regional activation level in response to image presentation

Specify type of analysis: ☐ Whole brain ☒ ROI-based ☐ Both

Anatomical location(s)	The functional localizer (fLoc) data was used to create contrast maps (voxel-wise t-statistics) of responses to specific object categories, and region boundaries were then manually drawn on inflated surface maps by identifying contiguous regions of high contrast in the expected cortical location, and thresholding to include all vertices with contrast > 0 within that boundary. Early visual ROIs were defined manually using retinotopic mapping data on the cortical surface. Surface-defined regions were projected back to fill in voxels within the gray matter ribbon.
Statistic type for inference (See Eklund et al. 2016)	Region-wise image responses were then calculated by averaging the voxel-wise beta response maps over all voxels within a given region.

Correction | We used permutation test to calculate the p-values.

Models & analysis

n/a	Involved in the study
☒	☐ Functional and/or effective connectivity
☒	☐ Graph analysis
☐	☒ Multivariate modeling or predictive analysis

Multivariate modeling and predictive analysis | We selected natural images based on the feature-weighted receptive field encoding model, and generated synthetic images based on NeuroGen framework. The encoding model has AlexNet as a multi-scale feature extractor, uses Gaussian pooling filter for dimension reduction and a ridge regression as readout. NeuroGen contains the encoding model described above and a image generator BigGAN, which takes in a noise vector and a class vector and outputs an image.
